# Genome-Wide Chromatin Analysis of FFPE Tissues Using a Dual-Arm Robot with Clinical Potential

**DOI:** 10.3390/cancers13092126

**Published:** 2021-04-28

**Authors:** Syuzo Kaneko, Toutai Mitsuyama, Kouya Shiraishi, Noriko Ikawa, Kanto Shozu, Ai Dozen, Hidenori Machino, Ken Asada, Masaaki Komatsu, Asako Kukita, Kenbun Sone, Hiroshi Yoshida, Noriko Motoi, Shinya Hayami, Yutaka Yoneoka, Tomoyasu Kato, Takashi Kohno, Toru Natsume, Gottfried von Keudell, Vassiliki Saloura, Hiroki Yamaue, Ryuji Hamamoto

**Affiliations:** 1 Division of Molecular Modification and Cancer Biology, National Cancer Center Research Institute, Tokyo 104-0045, Japan; nikawa@ncc.go.jp (N.I.); kshozu@ncc.go.jp (K.S.); adozen@ncc.go.jp (A.D.); hmachino@ncc.go.jp (H.M.); ken.asada@riken.jp (K.A.); maskomat@ncc.go.jp (M.K.); 2 Cancer Translational Research Team, RIKEN Center for Advanced Intelligence Project, Tokyo 103-0027, Japan; 3 Artificial Intelligence Research Center, National Institute of Advanced Industrial Science and Technology, Tokyo 135-0064, Japan; mituyama-toutai@aist.go.jp; 4 Division of Genome Biology, National Cancer Center Research Institute, Tokyo 104-0045, Japan; kshirais@ncc.go.jp (K.S.); tkkohno@ncc.go.jp (T.K.); 5 Department of Obstetrics and Gynecology, Faculty of Medicine, The University of Tokyo, Tokyo 113-0033, Japan; kukitaa-gyn@h.u-tokyo.ac.jp (A.K.); soneke-gyn@h.u-tokyo.ac.jp (K.S.); 6 Department of Diagnostic Pathology, National Cancer Center Hospital, Tokyo 104-0045, Japan; hiroyosh@ncc.go.jp (H.Y.); nmotoi@ncc.go.jp (N.M.); 7 Second Department of Surgery, School of Medicine, Wakayama Medical University, Wakayama 641-0011, Japan; shin-8@wakayama-med.ac.jp (S.H.); yamaue-h@wakayama-med.ac.jp (H.Y.); 8 Department of Gynecology, National Cancer Center Hospital, Tokyo 104-0045, Japan; yone1228@belle.shiga-med.ac.jp (Y.Y.); tokato@ncc.go.jp (T.K.); 9 Molecular Profiling Research Center for Drug Discovery, National Institute of Advanced Industrial Science and Technology, Tokyo 100-8921, Japan; t-natsume@aist.go.jp; 10 Robotic Biology Institute, Inc., Tokyo 135-0064, Japan; 11 Memorial Sloan-Kettering Cancer Center, New York, NY 10065, USA; vonkeudg@mskcc.org; 12 Center for Cancer Research, National Cancer Institute, Bethesda, MD 20892, USA; vassiliki.saloura@nih.gov

**Keywords:** epigenetics, ChIP-seq, FFPE, H3K4me3, H3K27ac, CTCF, robotics

## Abstract

**Simple Summary:**

Formalin-fixed paraffin-embedded (FFPE) specimens, which are pathological specimens of human tissues, are of high clinical value because they are associated with clinical information such as drug sensitivity and side effects and exist in huge numbers worldwide. However, the quality of DNA and RNA extracted from FFPE specimens is generally poor, and it is still difficult to perform ChIP-seq. Here, we describe an experimental procedure for FFPE ChIP-seq called RCRA ChIP-seq that allows identification of the genome-wide distributions of key histone modifications and binding sites of the insulator transcription factor CTCF. We have also succeeded in obtaining accurate and stable results even for the analysis of a large number of FFPE samples by using an industrial robot. Thus, routine ChIP-seq analysis of FFPE specimens could lead to new epigenomic mechanisms in various diseases.

**Abstract:**

Although chromatin immunoprecipitation and next-generation sequencing (ChIP-seq) using formalin-fixed paraffin-embedded tissue (FFPE) has been reported, it remained elusive whether they retained accurate transcription factor binding. Here, we developed a method to identify the binding sites of the insulator transcription factor CTCF and the genome-wide distribution of histone modifications involved in transcriptional activation. Importantly, we provide evidence that the ChIP-seq datasets obtained from FFPE samples are similar to or even better than the data for corresponding fresh-frozen samples, indicating that FFPE samples are compatible with ChIP-seq analysis. H3K27ac ChIP-seq analyses of 69 FFPE samples using a dual-arm robot revealed that driver mutations in EGFR were distinguishable from pan-negative cases and were relatively homogeneous as a group in lung adenocarcinomas. Thus, our results demonstrate that FFPE samples are an important source for epigenomic research, enabling the study of histone modifications, nuclear chromatin structure, and clinical data.

## 1. Introduction

Epigenetic alterations are crucial for the pathogenesis of human diseases, including cancer [1,2,3,4]. Chromatin immunoprecipitation coupled with next-generation sequencing (ChIP-seq) is a powerful technique to identify the genome-wide distribution of histone modifications and binding sites of transcription factors (TFs). The principles of ChIP-seq data analysis are similar to those of the corresponding microarray-based methods, such as mapping, enriched region identification, motif analysis, and integrated analysis of other datasets such as RNA-seq, but differ in that it can comprehensively analyze the entire genomic region. Since the development of the ChIP-seq method by Albert et al. [5] and Robertson et al. [6], various improvements have been suggested [7,8] but no standard experimental procedure has been established. In addition, there is a need to establish a simple procedure to enable high-throughput analysis due to the difficulty of processing multiple samples. Furthermore, ChIP-seq has been used on cultured cells and fresh-frozen (FF) tissues but rarely on clinically available samples, including formalin-fixed paraffin-embedded (FFPE) tissues. Owing to prolonged formalin fixation, FFPE tissues are heavily crosslinked and deteriorate over time, making it challenging to obtain a sufficient quantity of soluble chromatin required for ChIP-seq.

Preventing hydrolysis during prolonged storage minimizes protein degradation in FFPE samples [9]. Furthermore, techniques in heat-induced antigen retrieval have been widely used in the immunohistochemical staining of FFPE sections [10], suggesting that well-controlled heat treatments of clinically available FFPE tissues effectively retains antigenicity and crosslinked chromatin. Several studies have reported improvements in the extraction efficiency of soluble chromatin from FFPE samples using modified methods [11,12,13,14,15]. However, whether ChIP-seq using FFPE samples can detect bona fide histone modifications and binding sites of TFs remains to be fully understood. In this study, we describe a procedure employing reverse crosslinking to retrieve antigens and solubilize chromatin for ChIP-seq (RCRA ChIP-seq). Using these defined conditions, we identified histone modifications, such as H3K4me3 and H3K27ac, and binding sites of the insulator protein CTCF, a key TF that regulates nuclear chromatin structure and gene expression. Importantly, the RCRA ChIP-seq datasets of FFPE specimens were similar to the data obtained from freshly prepared samples of the matched tumors. Having established this novel RCRA ChIP-seq procedure, we performed H3K27ac ChIP-seq for 69 lung adenocarcinomas (LUADs) using a dual-arm robot, revealing that driver mutations in EGFR were epigenetically distinguishable from pan-negative cases. Overall, these results indicate that FFPE specimens are compatible for epigenetic research.

## 2. Materials and Methods

### 2.1. Clinical Materials

FFPE samples of hepatocellular carcinoma (HCC) and the matched frozen tissues were obtained from collections at Wakayama Medical University. FFPE samples of ovarian clear-cell carcinoma (OCCC) and the matched frozen tissues were obtained from collections at the University of Tokyo Hospital (summarized in Supplementary Table S1). For the purpose of comparison between archival clinical specimens and FF samples in a controlled fashion, fresh ovarian cancer tissues were obtained from the National Cancer Center Hospital (NCCH) and, in some cases, quick-frozen followed by fixation for one week in 10% buffered formalin before further processing to prepare FFPE blocks [12]. FFPE samples of LUAD were obtained from collections at the NCCH (clinical information is available in Supplementary Table S2). FFPE blocks were stored at room temperature in dry, dark conditions. All tissues were stained with hematoxylin and eosin (H&E) and reviewed by pathologists to confirm the histologic diagnosis. All methods were performed in accordance with the ethical guidelines for medical and health research involving human subjects. For the use of specimens in this research, informed consent was obtained from all patients, and the study was approved by the institutional review boards of Wakayama Medical University (871), the University of Tokyo Hospital (G0683-17), and the NCCH (2005-109, 2016-496).

### 2.2. RCRA ChIP-seq

**Figure 1:**
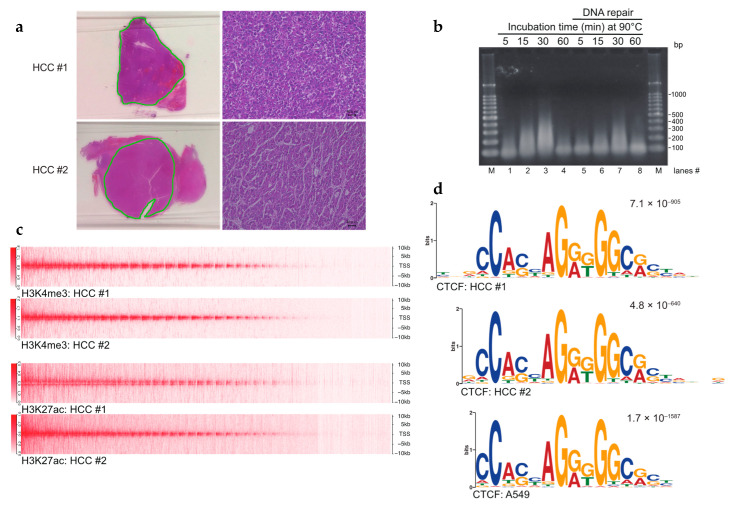
Establishment of the FFPE ChIP-seq procedure. (**a**) Hematoxylin and eosin (H&E) staining of hepatocellular carcinoma (HCC) tissues. Dissected tissues utilized for FFPE ChIP-seq are underlined in green. Higher magnification images (scale bars, 40 µm) are shown on the right sides. (**b**) Controlled heat-treatment of FFPE samples yielded solubilized chromatin. Sectioned FFPE tissues were treated with an overnight incubation at 65 °C followed by indicated time points at 90 °C. Reversed-crosslinked DNAs were repaired in lanes 5–8. Purified DNAs were amplified by whole genome amplification (WGA) and subjected to 1% agarose gel electrophoresis. M: 100-bp DNA ladders. (**c**) Heat map (upper half) showing H3K4me3 enrichment at TSSs ± 10 kb in HCCs. Heat map (lower half) for H3K27ac enrichment. (**d**) Sequence logos of CTCF ChIP-seq in HCCs. The top motif identified by MEME-ChIP, algorithms for de novo motif discovery, is shown. E-values, an estimate of the expected number of motifs with the given log-likelihood ratio, are shown on the upper right. Sequence logos in the A549 cell line were obtained from GSE30263.

FFPE tissues (8-µm thickness) were sectioned using Microtome (Leica, RM2165), mounted on membrane slides (Leica, 11505158), and dried at 37 °C overnight. If not processed immediately, the membrane slides on which the samples were mounted were stored at room temperature in the dark. The sections were washed with xylene three times and then rehydrated in an ethanol/water series (100/0, 95/5, 80/20, 70/30, 50/50, 20/80, 0/100). Each sample was macrodissected in order to isolate only the desired tumor, and then transferred to 1.5 mL tubes and stored at −80 °C if necessary. The samples were heated for 60 min at 65 °C followed by 30 min at 90 °C in a 1 mL of 1% sodium dodecyl sulfate (SDS) buffer containing 50 mM Tris-HCl (pH 8.0) and 10 mM EDTA (pH 8.0) with mixing in a Thermo shaker (1200 rpm; Chiyoda Science, MS-100). Note that 1 mL of SDS buffer was added to a sample with an approximate area of 250 mm^2^. The tissue pellets were obtained by centrifugation and resuspended with 250 µL of ChIP buffer containing 50 mM Tris-HCl (pH 8.0), 150 mM NaCl, 1% Triton X-100, 0.5% IGEPAL CA-630, 5 mM EDTA (pH 8.0), 1 mM phenylmethanesulfonyl fluoride (PMSF; Sigma-Aldrich, P7626), and 1× Protease Inhibitor Cocktail (1× PIC; Cell Signaling Technology, #7012). Samples were sonicated using Bioruptor II (BM Equipment, BR2006A) for 30 min at high 30-s ON and 30-s OFF cycles to generate DNA fragments of approximately 300 base pairs on average. Each antibody was added into the solubilized chromatin, briefly mixed, and incubated in an ultrasonic water bath (BM Equipment, BR2006A) for 40 min at low 90-s ON and 30-s OFF cycles. For H3K27ac ChIP, we added 10% SDS to final 1% SDS in ChIP buffer. After centrifugation, supernatants were incubated with 2 µL of FG Beads HM Protein G (Tamagawa Seiki, TAB8848N3173) for 30 min at 4 °C with rotation. Beads were washed twice with 800 µL of ChIP buffer, then once with 800 µL of wash buffer (50 mM Tris-HCl (pH 8.0), 300 mM NaCl, 1% Triton X-100, 0.1% SDS, 0.1% Na-deoxycholate, and 5 mM EDTA (pH 8.0)) and 800 µL of LiCl buffer (50 mM Tris-HCl (pH 8.0), 250 mM LiCl, 1% Triton X-100, 0.5% Na-deoxycholate, and 5 mM EDTA (pH 8.0)), each wash consisting of a 10-s mixing and 1 min collection on a magnetic rack. Immunoprecipitated chromatin was eluted with 150 µL of ChIP elution buffer (Cell Signaling Technology, #7009) for 30 min at 65 °C, and reverse-crosslinked by adding 6 µL of 5 M NaCl (Cell Signaling Technology, #7010) and 2 µL of Proteinase K (New England Biolab, P8107S) for ~16 h at 65 °C. DNA size was confirmed by 1% agarose electrophoresis (FUJIFILM Wako, 316-06071) using input samples. DNA was purified by QIAquick PCR Purification Kit (Qiagen, 28106) or Agencourt AMPure XP (Beckman Coulter, A63881) according to manufacturer instructions. The purified DNA samples were repaired by using PreCR Repair Mix (New England Biolabs, M0309) according to manufacturer instructions. In Figure 1b, we amplified DNAs with SeqPlex Enhanced DNA Amplification Kit (Sigma-Aldrich, SEQXE). The size determination and quantification of DNA was done by using Agilent 2100 Bioanalyzer or 4200 TapeStation (Agilent Technologies). The antibody information, library preparations, and Illumina sequencers used in this study were described in Supplementary Table S1.

### 2.3. Maholo RCRA ChIP-Seq

For the purpose of ChIP-seq using FFPE tissues of LUAD, the LabDroid Maholo (Robotic Biology Institute, CSDA10F) performed the FFPE ChIP-seq procedure as briefly described in Supplementary Figure S7 (see Supplementary Movie S1). The following equipment was installed in and around the Maholo: CO_2_ incubator (Astec, APC-30D), cool incubator (Mitsubishi electric engineering, CN-25D), rotating mixer (Nissinrika, NRC-20D), microtube mixer (Tomy digital biology, MT-400), deep freezer (Twinbird, SC-DF25), high-speed refrigerated microcentrifuge (Tomy digital biology, MX-307), suction pump (TMI, SP30), cooling dry bath (As one, EC-40RA), and thermostatic bath (As one, CB-100A). We used 2 µg of H3K27ac antibody (Abcam ab4729, lot GR3305164-1) per ChIP reaction in the presence of 1% SDS containing ChIP buffer described in the RCRA ChIP-seq method section. DNA libraries were prepared using KAPA HyperPlus Library Preparation Kit (Kapa Biosystems, KK8514) according to manufacturer instructions. All DNA libraries were amplified with 15-cycle PCR and sequenced on an Illumina NovaSeq 6000 platform.

### 2.4. Fresh and FF Tissue ChIP-Seq 

Chromatin immunoprecipitation was performed according to the manufacturer’s instructions (Cell Signaling Technology, #9003) with minor modifications [16]. For the FF tissues ChIP-seq, dissected sections of 8-µm thickness for tumor specimens were crosslinked with 1% formaldehyde (Sigma-Aldrich, 252549) in 1× PBS (-) (FUJIFILM Wako, 293-72601) for 10 min at room temperature. Crosslinking was quenched by the addition of 10x glycine solution (Cell Signaling Technology, #7005) for 5 min at room temperature. Crosslinked tissues were washed with ice-cold 1× PBS (-). For the fresh tissues ChIP-seq, the samples were minced using a razor blade on ice and transferred to 1.5 mL tubes in ice-cold 1× PBS (-). Then, 37% formaldehyde was directly added to the final 1% concentration and incubated for 10 min at room temperature. Crosslinking was quenched as described above. Crosslinked tissues were resuspended with 1 mL of 1× Buffer A (Cell Signaling Technology, #7006) with 1 mM PMSF and 1× PIC, and incubated on ice for 10 min. After centrifugation, nuclei pellets were resuspended with 1 mL of Buffer B (Cell Signaling Technology, #7007) with 0.5 mM dithiothreitol (DTT; Cell Signaling Technology, #7016). After centrifugation, nuclei pellets were resuspended with 100 µL of Buffer B with 0.5 mM DTT. Then, 1 µL of micrococcal nuclease (Cell Signaling Technology, #10011) was added into the nuclei mixture and incubated for 20 min at 37 °C with frequent mixing (800 rpm). DNA digestion was stopped by adding 20 µL of 0.5 M EDTA (pH 8.0). After centrifugation, nuclei pellets were resuspended with 200 µL of ChIP buffer as described in the RCRA ChIP-seq method section. Sample sonication, immunoprecipitation, reverse-crosslinking, DNA clean-up, and DNA library preparation were described in the RCRA ChIP-seq method section, except the preparation of repaired DNA was omitted. The antibody information, library preparations, and Illumina sequencers used in this study were described in Supplementary Table S1.

### 2.5. Bioinformatic Analysis 

The CTCF ChIP-seq datasets of A549 cell lines were obtained from GSE30263, a part of the ENCODE Project [17]. Sequenced reads from ChIP-seq experiments were mapped to the hg38 version of the human genome with Bowtie (v2.2.9) and parameters local [18]. Duplicate reads were removed by Samtools (v1.3.1). Heatmaps were generated with NGSplot (v2.63) [19]. Enriched regions (ERs) were identified with MACS (v1.4.2) with the relevant input as control [20]. Unless specified, MACS peak calling was done by default setting (*p*-value cutoff: 1 × 10^−5^). Peak calling was done by AIControl without Input [21] when performing the H3K27ac ChIP-seq of LUAD. The normalized ERs were visualized with the Integrative Genomics Viewer, IGV (v2.3.91) [22]. Gene annotations were obtained from Ensemble (v.86). Unless specified, the peaks were annotated with the genes within 5-kb upstream and downstream of the ERs by using ChIPpeakAnno (v3.10.2) [23]. Motif analyses were done by MEME-ChIP (v5.1.1) with ERs (100 bp window) around peak summit using the following command; meme. /seqs-centered -oc meme_out -mod zoops -nmotifs 3 -minw 6 -maxw 30 -bfile./background -dna -searchsize 100000 -time 5082 -revcomp –nostatus [24]. To assess the genome-wide similarity of ChIP-seq datasets, we obtained the read coverages for the entire genome divided into 10-kb bins. Pearson correlation coefficients were computed by deepTools2 with read processing option, —removeOutliers [25]. Overlapping peaks of ERs between different ChIP-seq experiments were obtained by using the “findOverlapsOfPeaks” function under the default setting in ChIPpeakAnno. This setting counts the peaks as the minimally involved peaks in any group of connected/overlapped peaks. Differential binding analysis with H3K27ac ChIP-seq of LUAD was performed using edgeR with TMM normalization implemented in DiffBind (v2.4.8) [26]. Principal components analysis (PCA) from a normalized read count matrix of H3K27ac ChIP-seq was performed on the basis of singular value decomposition (SVD). For PCA implementation, we used BiocSingular (v1.2.2) and PCAtools (v1.2.0) R packages. The Kyoto Encyclopedia of Genes and Genomes (KEGG) over-representation test was done by using the clusterProfiler R package (v3.14.3) under the default setting on the basis of hypergeometric distribution [27]. For a volcano plot representation, we used the EnhancedVolcano (v1.4.0) R package. Hierarchical clustering analysis was done using Euclidean distance and Ward’s linkage method (ward.D2) with options (gene ranking option: SD_Rank, the number of ERs selected: 100%, cluster number: 2) implemented in multiClust R package (v1.16.0) [28] and visualized with Java TreeView (v1.1.6r4) [29]. 

### 2.6. Statistical Analysis 

All data and statistical analyses were performed in Excel and R as described in Methods. We used GraphPad Prism (GraphPad Software, Inc. San Diego, CA, USA; v7). All image data were analyzed by using ImageJ (v1.51j8). The significance values and sample size in the respective figures were described in the corresponding results or figure legends sections. Correlations were conducted using a Pearson correlation coefficient. *p*-values are indicated in the figures and figure legends.

## 3. Results

### 3.1. Chromatin Solubilization of FFPE Tissues

The major challenges associated with performing ChIP-seq using FFPE samples in clinical studies are as follows: (1) the requirement for large quantities of tissues from limited resources, such as human tumor specimens; (2) low chromatin yield due to extensive crosslinking of tissue samples; and (3) the complicated protocol for large-scale epigenetic studies with high reproducibility. Thus, we wanted to understand the optimal heating conditions required to retrieve antigenic protein(s) of interest while retaining moderately crosslinked chromatin. We incubated deparaffinized and rehydrated HCC tissue sections at 65 °C overnight, a typical condition in the reverse-crosslinking step (Figure 1a, Supplementary Figure S1a, Step 3). We obtained DNA fragments that were ~100 bp shorter than that in a mononucleosome, implying insufficient reverse crosslinking. Strikingly, overnight incubation at 65 °C followed by that at 90 °C resulted in a marked increase in the levels of ~200–300 bp long DNA fragments in a time-dependent manner (Figure 1b, lanes 1–3 and 5–7), suggesting the utility of incubating samples at 90 °C for 30 min to obtain solubilized chromatin. However, incubation at 90 °C for 60 min resulted in the considerable loss of DNA, indicating DNA degradation via the depurination of nucleic acids [30] (Figure 1b, lanes 4 and 8). Since the 90 °C heat treatment was found to be important, in the following experiments, the time was reduced to 60 min at 65 °C and then 30 min at 90 °C (see Methods). 

A key factor for ChIP-seq is obtaining sufficient amounts of chromatin-containing lysates, approximately more than several hundred nanograms of ChIP-compatible chromatin per reaction. To determine the minimum volume of FFPE tissues required for ChIP-seq, we compared the yields of chromatin from different volumes of FFPE tissue sections using the heating condition described previously herein. We found that ~250 mm^2^ and 8-µm thick HCC tissues, typically in 1–2 thin sections, yielded enough soluble chromatin with variability, depending on the total cellularity of the HCC samples (approximately 400 ng to 2 µg, Figure 1a and Supplementary Figure S1b). Notably, increasing the amount of FFPE tissue sections resulted in poor recovery of chromatin, suggesting the requirement of an optimum ratio of FFPE tissue sections and solubilization buffer (Supplementary Figure S1c). To understand the relationship between the type of tumor and its corresponding chromatin yield, we used the same protocol for OCCC tissues. We obtained comparable amounts of soluble chromatin in OCCC (Supplementary Figures S1b and S2a). Overall, these results indicate that a heat treatment followed by standard sonication is sufficient for obtaining ChIP-compatible chromatin using limited FFPE samples.

### 3.2. FFPE ChIP-Seq 

To investigate the compatibility of chromatin prepared using this procedure, we performed ChIP with anti-H3K4me3, anti-H3K27ac, and anti-CTCF antibodies under previously reported conditions [16] together with the DNA repair process to recover damaged DNA molecules (Supplementary Figure S1a). This repair process has been shown to correct for FFPE-induced DNA damage [31]. RCRA ChIP-seq profiling of the active chromatin marks showed the enrichment of H3K4me3 and H3K27ac around transcription start sites (TSSs) in HCC samples (Figure 1c). Moreover, motif discovery tools, like MEME-ChIP [24], revealed that the binding sites of CTCF were similar to the binding sites in A549 cells obtained using the ENCODE project [17] (Figure 1d). Of note, the CTCF binding sites are characterized by a specific motif, highly conserved in vertebrates [28]. To demonstrate the applicability of the RCRA ChIP-seq procedure to other cancer types in different hospitals, we mapped the active chromatin marks H3K4me3 and H3K27ac, and binding sites of CTCF in OCCC samples (Supplementary Table S1 and Figure S2a). There was a significant enrichment of active chromatin marks at the TSSs and the CTCF consensus motif (Supplementary Figure S2b,c). Thus, the RCRA ChIP-seq procedure was capable of identifying the genome-wide distribution of specific histone modifications related to transcriptionally active marks and the CTCF insulator protein.

### 3.3. Epigenetic Status of Oncogenes 

**Figure 2:**
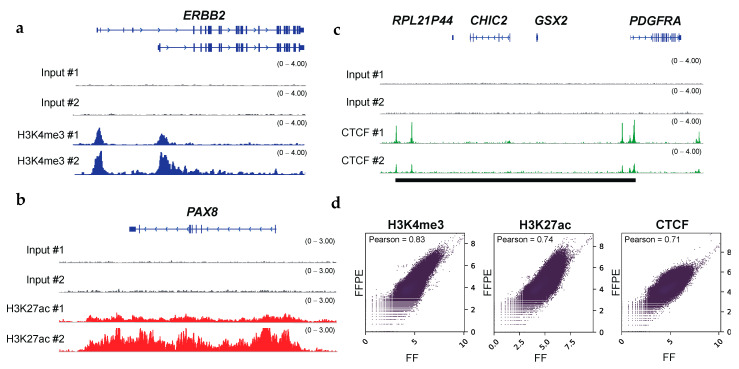
FFPE ChIP-seq with clinical specimens obtains the histone modification status of oncogenes and the binding status of the transcription factor CTCF. (**a**) IGV tracks (blue) of H3K4me3 peaks at ERBB2 locus, illustrating alternative promoter activities of oncogenes in OCCCs. (**b**) IGV tracks (red) of H3K27ac peaks at PAX8 locus, illustrating broad enhancer regions at oncogenes. (**c**) IGV tracks (green) of CTCF peaks at PDGFRA locus. Putative insulated neighborhoods are shown in black bars. Input controls are shown as gray. (**d**) Scatter plots showing the correlation between ChIP-seq datasets with FFPE and the matched FF in OCCCs. The read coverages for entire genomic regions (bin sizes: 10-kb) were utilized. The results for H3K4me3 (left), H3K27ac (middle), and CTCF (right) are shown. Pearson correlation coefficients are shown on the upper left.

H3K4me3 profiling is commonly used to identify active promoters closely located at TSSs [32]. In the human genome, the majority of protein-coding genes are known to be regulated by multiple promoters that initiate the transcription of different gene isoforms [33,34]. The choice of alternative promoters is one of the signatures for context-specific transcriptional regulation and the malignant transformation of cells [35,36,37]. Notably, our H3K4me3 ChIP-seq using FFPE tissues identified alternative promoters of *ERBB2*, also known as the *HER2* oncogene in OCCC [35] (Figure 2a). We also identified alternative promoter activation for *SEPT9*, a biomarker for a variety of cancers [38] (Supplementary Figure S3a). Given that tumor-specific enhancers and super-enhancers have frequently been identified using H3K27ac ChIP-seq [39,40], we wanted to identify enhancer regions of the previously reported oncogenes. Indeed, our H3K27ac ChIP-seq using FFPE tissues showed broad H3K27ac marks within several oncogenes, such as *PAX8*, *MYC*, *UCA1*, and *FOSL2*, in OCCC samples [41,42] (Figure 2b and Supplementary Figure S3b). The CTCF insulator protein is essential for organizing the genome into topologically associated domains [43,44]. The loss of CTCF boundaries causes inappropriate enhancer–promoter interactions and dysregulated local gene expression in cancer. Multiple oncogenes, such as *PDGFRA, TAL1*, and *LMO2*, are transcriptionally activated after the perturbation of CTCF–CTCF interactions at defined loci [45,46,47]. In this study, RCRA ChIP-seq detected CTCF binding sites that could act as boundary elements (Figure 2c and Supplementary Figure S3c). Overall, these analyses demonstrated that RCRA ChIP-seq reliably captures the epigenetic profiles of oncogenes. Of note, we have performed ChIP-seq using FFPE samples of different carcinomas from three different hospitals, and the quality of the results obtained is comparable between the different hospitals in terms of the number of peaks called by MACS (see Supplementary Table S1).

### 3.4. Comparisons with Fresh and FF Tissue ChIP-Seq 

**Figure 3:**
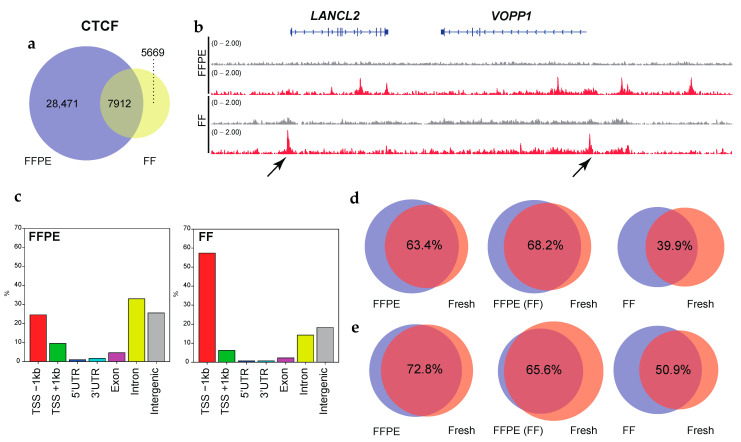
The FFPE samples retain accurate CTCF binding. (**a**) Venn diagrams showing the overlap of CTCF binding between FFPE and the matched FF in OCCC. (**b**) IGV tracks of CTCF peaks (red), comparing FFPE and the matched FF in OCCC. Input controls are shown as gray. Non-concordant peaks are shown by the arrows. (**c**) Bar graphs display the genomic distribution of the CTCF binding sites in FPPE (left side) and the matched FF (right side) tissues. (**d**) Venn diagrams showing the overlap of CTCF binding between FFPE and fresh (left), pre-frozen FFPE and fresh (middle), and FF and fresh (right) in endometrial endometrioid adenocarcinoma (EEA). (**e**) Same as (**d**) but for cervical carcinosarcoma (CC). The *p*-value cutoff for the MACS peak calling in panels d and e is 1 × 10^−9^. The overlap ratios (%) are shown.

Given that ChIP-seq using FF tissues is associated with better signal-to-noise ratios compared to that using FFPE tissues, FF ChIP-seq has usually been used as validation datasets [12,14,15]. We analyzed the quality of FFPE ChIP-seq datasets compared with those derived from the matched FF tumor specimens for OCCCs. Pearson correlations for pairwise comparisons were 0.83, 0.74, and 0.71 for H3K4me3, H3K27ac, and CTCF, respectively, indicating a quantitative correlation between the FFPE and FF ChIP-seq datasets (Figure 2d). In RCRA ChIP-seq, MACS peak caller identified 29,635 peaks using the default setting (*p*-value cutoff: 1 × 10^−5^) and 18,341 peaks (*p*-value cutoff: 1 × 10^−9^) for the H3K4me3 mark. Furthermore, we also obtained 48,431 and 37,935 peaks for H3K27ac and CTCF, respectively, using the default parameters (Supplementary Table S1). Although these peaks significantly overlapped with those found in the FF ChIP-seq datasets (Supplementary Figure S4), we noticed that non-concordant peaks for CTCF, albeit robust, (Figure 3a,b and Supplementary Figure S5), were highly enriched near TSSs in FF tissues (Figure 3c). Given that CTCF primarily binds to intergenic and intronic regions [48,49,50], these results prompted us to systematically investigate the effects of 10% formalin fixation, typically utilized in the protocol of FFPE tissue preparation on RCRA ChIP-seq. To this end, we prepared chromatin from two independent clinical tumor tissues, endometrial endometrioid adenocarcinoma (EEA) and cervical carcinosarcoma (CC), in a controlled manner as follows: (1) fresh, (2) FF, (3) FF followed by FFPE, and (4) FFPE.

To mimic the effects on chromatin in archival clinical specimens, we fixed the tissues for 1 week (168 h) for FFPE preparations as reported [12]. As a consequence, the CTCF binding peaks for FFPE samples significantly overlapped (63.4% in EEA, 72.8% in CC) with the peaks for fresh samples (Figure 3d,e, left panels). The degree of overlap decreased between the FF (39.9% in EEA, 50.9% in CC) and the fresh samples (right panels). Freezing FFPE samples in advance did not affect the positioning of the peaks (68.2% in EEA, 65.6% in CC) (middle panels). Again, we observed that CTCF binding sites using FF tissues tended to accumulate near TSSs compared to that using FFPE (Supplementary Figure S6). These results indicated that RCRA ChIP-seq retained the binding sites for CTCF.

### 3.5. Large-Scale ChIP-Seq Using a Dual-Arm Robot

**Figure 4:**
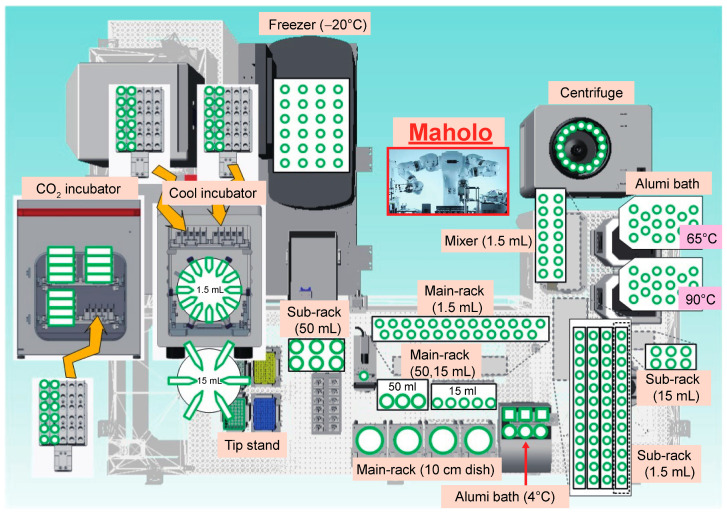
General arrangement drawing for the experimental devices placed around the Maholo. The Maholo is centrally located. Note that the magnetic stand and the refrigerated centrifuge are located in the middle and upper right corner, respectively.

Having established the protocol for RCRA ChIP-seq, we performed a large-scale RCRA ChIP-seq analysis to understand the epigenetic landscape in cancer patients. We used a double-armed industrial robot, the LabDroid system named ‘Maholo’, capable of performing basic wet-lab experiments (Figure 4 and Supplementary Movie S1). Since Maholo can reproduce human tasks without the assistance of action-specific jigs, highly sophisticated workflows can be programmed using a combination of pre-defined motions [51]. We divided the protocol for RCRA ChIP-seq into three steps as follows: chromatin solubilization, ChIP followed by DNA clean-up, and DNA library preparation. Each process (excluding handling the ultrasound water bath) was fully automated (Supplementary Figure S7).

**Figure 5:**
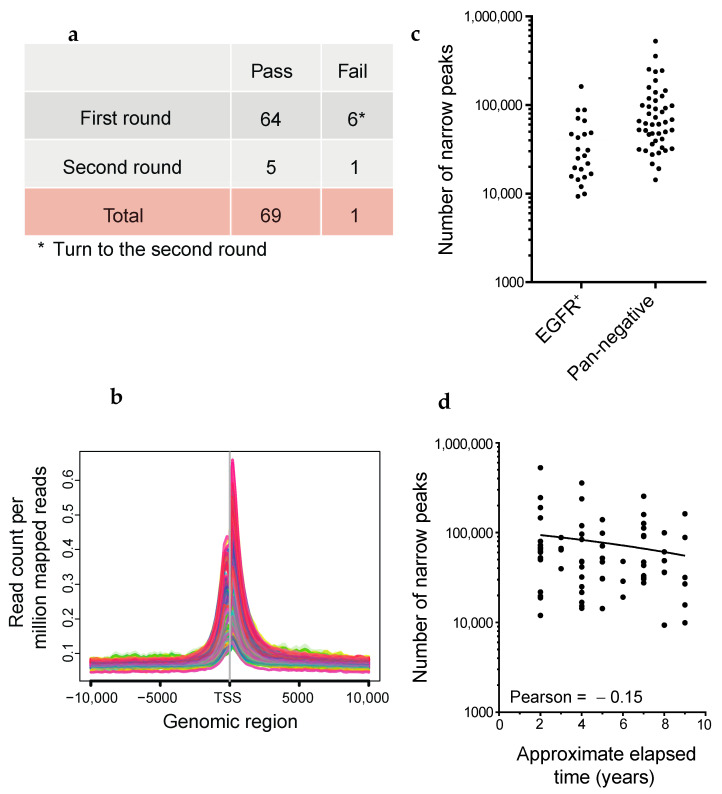
Quality evaluation of FFPE ChIP-seq analysis using the dual-arm robot. (**a**) Experimental strategy. The six cases denoted in the asterisk performed the second round of FFPE ChIP-seq in LUADs. (**b**) Average profile plots showing H3K27ac enrichment around TSSs ±10 kb. The y-axis represents read count per million mapped reads. (**c**) Dot plots showing the number of called peaks generated by AIControl (see Methods). EGFR-mutant positive and pan-negative cases are shown on the left and right sides, respectively. (**d**) Scatter plot showing the number of called peaks in every elapsed period of FFPE tissues. Pearson correlation coefficient is shown.

LUAD is a type of lung cancer that has mutations in oncogenic drivers such as *EGFR, KRAS, BRAF,* and *HER2*, gene fusions including *ALK, RET, ROS1, NRG1,* and *BRAF*, and the skipping of *MET* exon 14 [52,53]. To understand the genes that depend on enhancers for their role in LUAD tumorigenesis, we programmed Maholo to perform H3K27ac ChIP-seq on 69 clinical FFPE samples of LUAD obtained from collections at the National Cancer Center Hospital in Japan (Figure 5a). Among these, 23 samples showed driver mutations in *EGFR*, while 46 samples had no identifiable driver mutations described previously herein (referred to as ‘pan-negative’ in this study) (Supplementary Table S2). Peak calling was performed by AIControl without Input [21]. We judged the quality of RCRA ChIP-seq based on the number of called peaks and H3K27ac enrichment at TSSs (Figure 5b,c), and repeated RCRA ChIP-seq when we obtained the apparently low number of peaks (Figure 5a). We observed a slight decrease in the number of called peaks with prolonged storage; this was consistent with the increase in DNA degradation in older FFPE tissue blocks [50] (Figure 5d). There was an average of 40,412 and 96,546 called peaks for EGFR mutation-positive and pan-negative samples, respectively, in accordance with the relatively more fresh FFPE tissue blocks in the latter cases (EGFR mutation: 5.48, pan-negative: 4.61 years on average, Figure 5c and Supplementary Table S2). IGV tracks showed the enrichment of H3K27ac in the NEAT1 and MALAT1 constitutively transcribed lncRNAs (Supplementary Figure S8). On a chromosome-wide scale, enriched regions (ERs) of H3K27ac marks were observed throughout; differential ERs (DERs; |log fold change (FC)| >1, false discovery rate (FDR) < 0.05) were also observed (Supplementary Figure S9a,b), suggesting that RCRA ChIP-seq is a genome-wide analysis.

**Figure 6:**
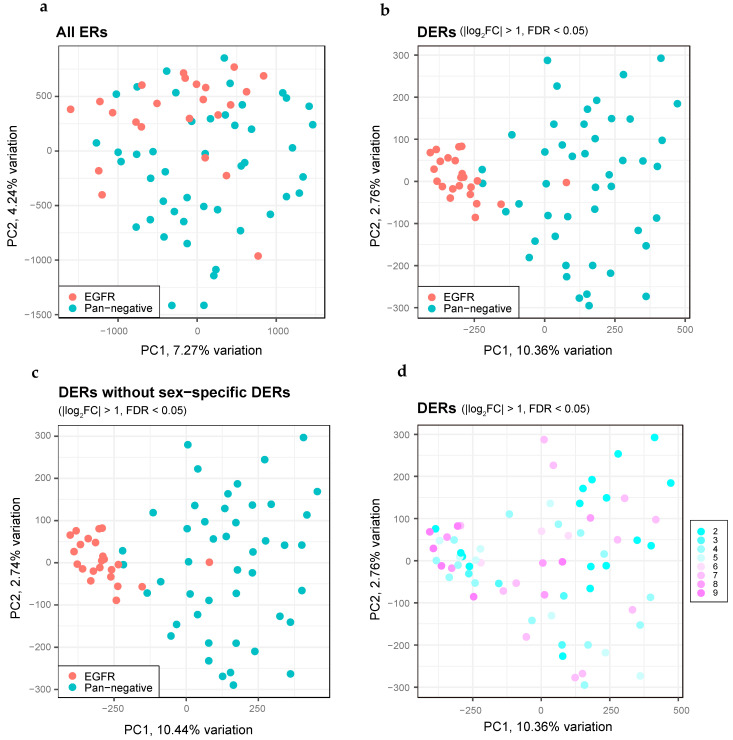
Distinct features of DERs between EGFR-mutant and pan-negative LUADs obtained by H3K27ac ChIP-seq. (**a**) PCA plots for all ERs, comparing EGFR-mutant (red dots, *n* = 23) and pan-negative (blue dots, *n* = 46) LUADs. (**b**) The same as panel (**a**) but showing PCA plots for DERs. (**c**) The same as panel (**b**) but we excluded DERs on the sex chromosomes. (**d**) The same as panel (**b**) but we highlighted the elapsed time (years) of each FFPE tissue in colors.

To elucidate the epigenetic differences among LUAD patients, we performed a principal component analysis of the H3K27ac ChIP-seq datasets. The EGFR mutation-positive LUAD profiles showed significant overlap in the ERs with those of the pan-negative cases, suggesting that a significant fraction of ERs were common irrespective of the status of driver mutations (Figure 6a). Importantly, DERs in EGFR mutation-positive LUADs constituted a dense cluster, in stark contrast to the DERs of the pan-negative cases (Figure 6b). Although DERs on the X chromosome were relatively enriched in EGFR mutation-positive LUADs (Supplementary Figure S9c,d), omission of the DERs on the sex-specific chromosomes did not change the cluster distribution (Figure 6c), suggesting that epigenetic alterations upon EGFR mutations in LUAD do not involve the sex chromosomes. Although age-related degradation of DNA was observed in the FFPE tissues, no bias was noted in the distribution of the clusters (Figure 6d).

**Figure 7:**
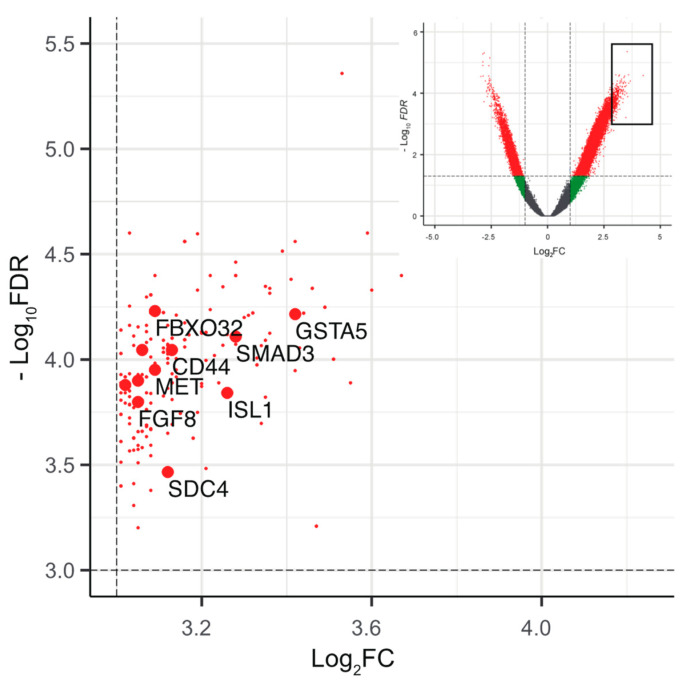
Epigenetic landscape of driver mutations in pan-negative LUADs. Partially enlarged volcano plots showing the top annotated DERs (log2FC > 3, −log10 FDR > 3, *n* = 208, red dots) in pan-negative cases. The annotated genes associated with the Kyoto Encyclopedia of Genes and Genomes (KEGG) pathway are highlighted in larger red dots (see Supplementary Table S4). The inset shows the entire plots to indicate the enlarged area (black square)**.**

Finally, to understand the epigenetics associated with pan-negative LUAD, we analyzed a common set of genes associated with the DERs in pan-negative cases (Supplementary Figure S10 and Table S3, see Methods). Among the top DERs (log_2_FC > 3, −log_10_FDR > 3, *n* = 208), many of the gene sets were enriched in cancer-related pathways, highlighting the genes critical for driving tumorigenesis in pan-negative LUAD (Figure 7 and Supplementary Tables S3 and S4).

## 4. Discussion

Here we demonstrated that RCRA ChIP-seq, a simple heat-based treatment of FFPE samples followed by ChIP-seq, is a reliable method to study genome-wide histone modifications and CTCF binding sites using a limited amount of sample. Notably, RCRA ChIP-seq yielded results concordant with those obtained using fresh samples from the same patients. There was reduced overlap in CTCF binding sites between data from FF and fresh tissue samples. Although the reason for this is not clear, non-specific binding of CTCF may be a result of changes in the three-dimensional structure of the genome during the freeze-thaw process. Alternatively, the DNA repair treatment during RCRA ChIP-seq may help to correct discordant peaks. Thus, to the best of our knowledge, this is the first report to demonstrate that FFPE sections faithfully retain genome-wide DNA-chromatin binding sites (see Figure 3d,e).

FFPE tissues are not normally used in molecular genetic analyses owing to extensive degradation of their nucleic acid content. In this study, RCRA ChIP-seq efficiently retrieved soluble chromatin from limited amounts of clinically available specimens that were immunoprecipitated using antibodies against H3K4me3, H3K27ac, and CTCF. Since ChIP-seq usually includes DNA fragmentation using a sonicator, the presence of partially degraded DNA might not be a problem [54]. Indeed, we obtained biologically relevant ChIP-seq datasets from OCCC and LUAD samples stored for 9 years (Figure 2, Supplementary Tables S1 and S2).

Although the protocol for RCRA ChIP-seq is analogous to that for EPAT-ChIP, Chrom-EXPE, and FiTAc-seq [13,14,15], including methods to reverse-crosslink the chromatin of FFPE tissues at 65 °C or higher temperatures, the controlled conditions in this technique, in terms of temperature and volume ratio of sample to buffer, extend the utility of ChIP-seq for targeting TFs (Supplementary Table S1, see Methods). Our RCRA ChIP-seq procedure also showed promise using an industrial robot that was fully automated, programmable, and capable of flexible movement on the dual-arm (7-axis each). We performed FFPE ChIP-seq on 69 LUAD samples with simultaneous processing using the Maholo system. The ChIP-seq procedure is relatively long and has never been executed by an industrial robot. Generally, robots equipped with specified peripheral items have limited use in the biological laboratory owing to the high cost for routine experiments. However, Maholo circumvents this problem since we only used the robot system equipped with a series of common lab tools found in ordinary wet laboratories (Figure 4). Thus, the Maholo system can be used for large-scale medical research with high reproducibility for methods previously published using a combination of pre-defined motions. Indeed, our system can be applied by having the robot implement a protocol that corresponds to the sample form. In fact, we have performed ChIP-seq analysis of more than 150 cases using frozen surgical tissue specimens (data not shown). Furthermore, it is intriguing to test whether using a robot will produce more reproducible results than doing it manually.

Integrated analyses of genetic and epigenetic datasets using machine learning algorithms have shown significant potential in current research [55,56]. Generally, large-scale omics datasets in cancer include, but are not limited to, the whole genome, transcriptome, and DNA methylation profiles. The FFPE ChIP-seq developed in this study contributes an additional layer of omics datasets, thereby integrating the genetics, transcriptomics, and phenomics of a biological system. The H3K27ac ChIP-seq of LUAD demonstrated that EGFR mutation-positive samples formed a dense cluster during principal component analysis as compared to that with pan-negative cases, thereby confirming that oncogenic driver mutations in EGFR are phenotypically uniform in patients with LUAD. This can help understand the favorable clinical response of EGFR-mutant LUAD patients treated with EGFR tyrosine kinase inhibitors [57,58,59]. It is intriguing to investigate the epigenetic status of other driver mutations, as well as pan-negative cases in LUAD, using large-scale FFPE ChIP-seq analysis.

However, this study has some limitations as follows: (1) high-quality antibodies are necessary for the success of FFPE ChIP-seq in terms of lot-to-lot variation, and (2) low genomic coverage of ChIP-seq signals owing to the small amounts of solubilized chromatin during ChIP. These limitations might be improved by the techniques to individually optimize chromatin solubilization for each sample. This idea is likely feasible when using dual-arm robots. In conclusion, this study reveals that clinical FFPE tissues are excellent sources to analyze the pathology and molecular biology of cancers using ChIP-seq. Moreover, large-scale clinical FFPE ChIP-seq is possible using an industrial robot, highlighting the utility of the protocol developed as a novel tool in epigenetic research.

## 5. Conclusions

We developed an experimental procedure for FFPE ChIP-seq, called RCRA ChIP-seq, and succeeded in identifying the genome-wide distribution of major histone modifications and the binding sites of the insulator transcription factor CTCF. We also succeeded in obtaining accurate and stable results even when analyzing a large number of FFPE samples using an industrial robot. Thus, our RCRA ChIP-seq method could be applied to elucidate new epigenomic mechanisms in various diseases.

## Data Availability

Data sharing is not applicable for this article.

## Supplementary Materials

The following are available online at https://www.mdpi.com/article/10.3390/cancers13092126/s1, Figure S1: Optimization for chromatin solubilization with clinical FFPE tissues, Figure S2: FFPE ChIP-seq with OCCCs, Figure S3: Tracking of epigenetic status for the individual oncogenes, Figure S4: Comparison of ChIP-seq using FFPE and FF samples, Figure S5: Examples of non-concordant peaks, Figure S6: Genomic distribution of the CTCF binding sites, Figure S7: Procedure for the Maholo ChIP experiment, Figure S8: IGV tracks for H3K27ac ChIP-seq at NEAT1 and MALAT1 loci, Figure S9: Chromosome distribution of H3K27ac-enriched regions, Figure S10: The majority of DERs are properly grouped, Table S1: Sample characteristics, antibody information, and ChIP-seq library statistics, Table S2: Clinical information of LUAD in this study, Table S3: The list of annotated DERs for H3K27ac ChIP-seq in LUADs (|log2 FC| > 2, FDR < 5 × 10^−4^, n = 2128), Table S4: The list of KEGG pathways associated with annotated DERs (log2FC > 3, FDR < 1 × 10^−3^, n = 120), Video S1: A LabDroid system is utilized for FFPE ChIP-seq. A representative robot movement is introduced with a speed up factor of 2.5.
