# Supplementary material for: Genome-Wide Chromatin Analysis of FFPE Tissues Using a Dual-Arm Robot with Clinical Potential
Source: Cancers (Basel). 2021 Apr 28;13(9):2126. doi: 10.3390/cancers13092126 (PMC8125448; doi:10.3390/cancers13092126)
Supplement: Supplementary file 1 [file cancers-13-02126-s001.zip › cancers-1178987-supplementary materials/cancers-1178987-supplementary materials.pdf]

cancers

MDPI

Supplementary Materials

Genome-Wide Chromatin Analysis of FFPE Tissues Using a Dual-Arm Robot with Clinical Potential

Syuzo Kaneko, Toutai Mitsuyama, Kouya Shiraishi, Noriko Ikawa, Kanto Shozu, Ai Dozen, Hidenori Machino, Ken Asada, Masaaki Komatsu, Asako Kukita, Kenbun Sone, Hiroshi Yoshida, Noriko Motoi, Shinya Hayami, Yutaka Yoneoka, Tomoyasu Kato, Takashi Kohno, Toru Natsume, Gottfried von Keudell, Vassiliki Saloura, Hiroki Yamaue and Ryuji Hamamoto

a

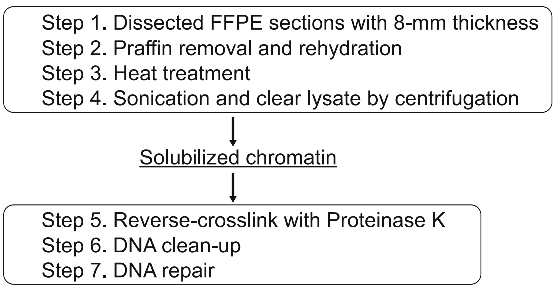

b

Tumor type	Sample ID	Tumor area (mm²)	Chromatin yield (ng)
HCC	1	226.4	2040
HCC	2	240.1	628
OCCC	1	177.7	378
OCCC	2	197.1	330

c

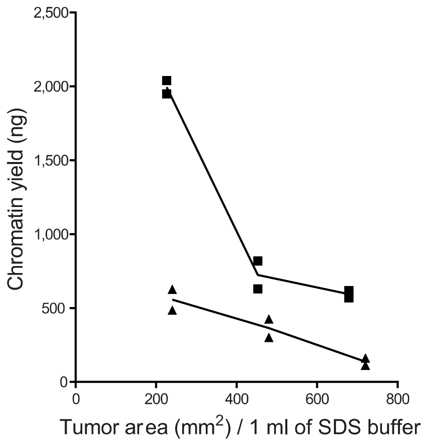

Figure S1. Optimization for chromatin solubilization with clinical FFPE tissues. (**a**) Schematic diagram of chromatin extraction method from clinical FFPE tissues. (**b**) Chromatin yield of FFPE ChIP procedure from HCC and OCCC tissues. The DNA amount purified from soluble fraction was measured. (**c**) Same as **b** but for increasing the amount of FFPE tissues of HCCs. Black square: Sample ID #1, black triangle: Sample ID #2.

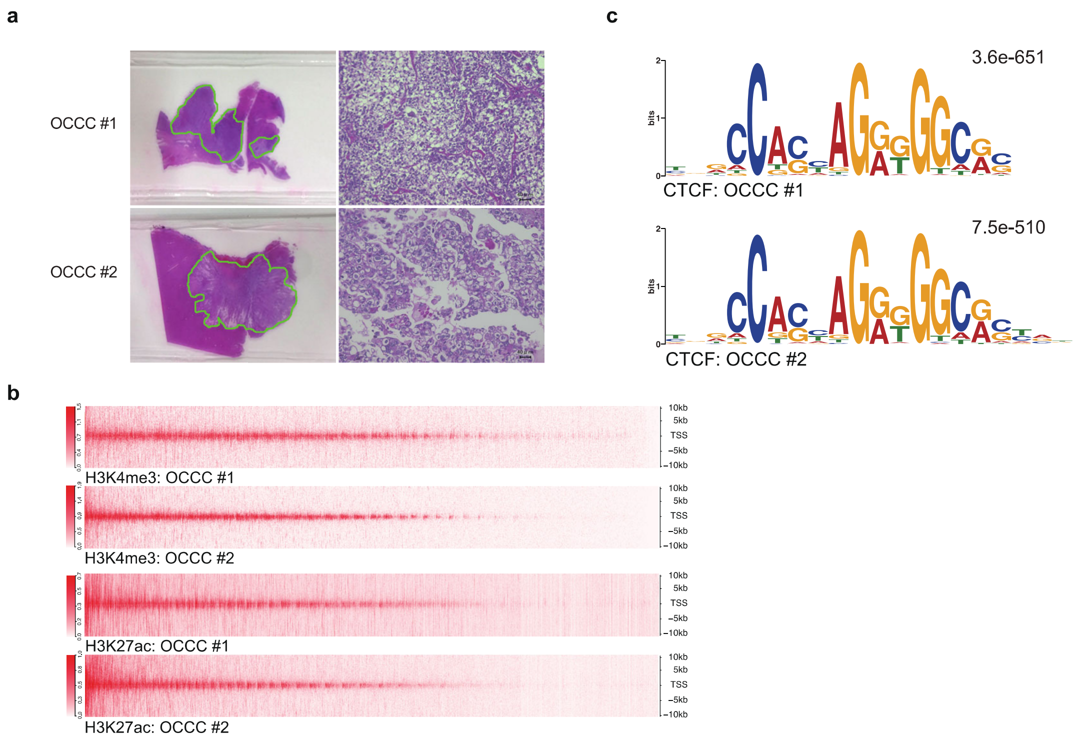

Figure S2. FFPE ChIP-seq with OCCCs. (**a**) H&E staining of ovarian clear-cell carcinoma (OCCC) tissues as with Figure 1a. (**b**) Heat map (upper half) showing H3K4me3 enrichment at TSSs ±10kb in OCCCs. Heat map (lower half) for H3K27ac enrichment. (**c**) Sequence logos in OCCCs as with Figure 1d.

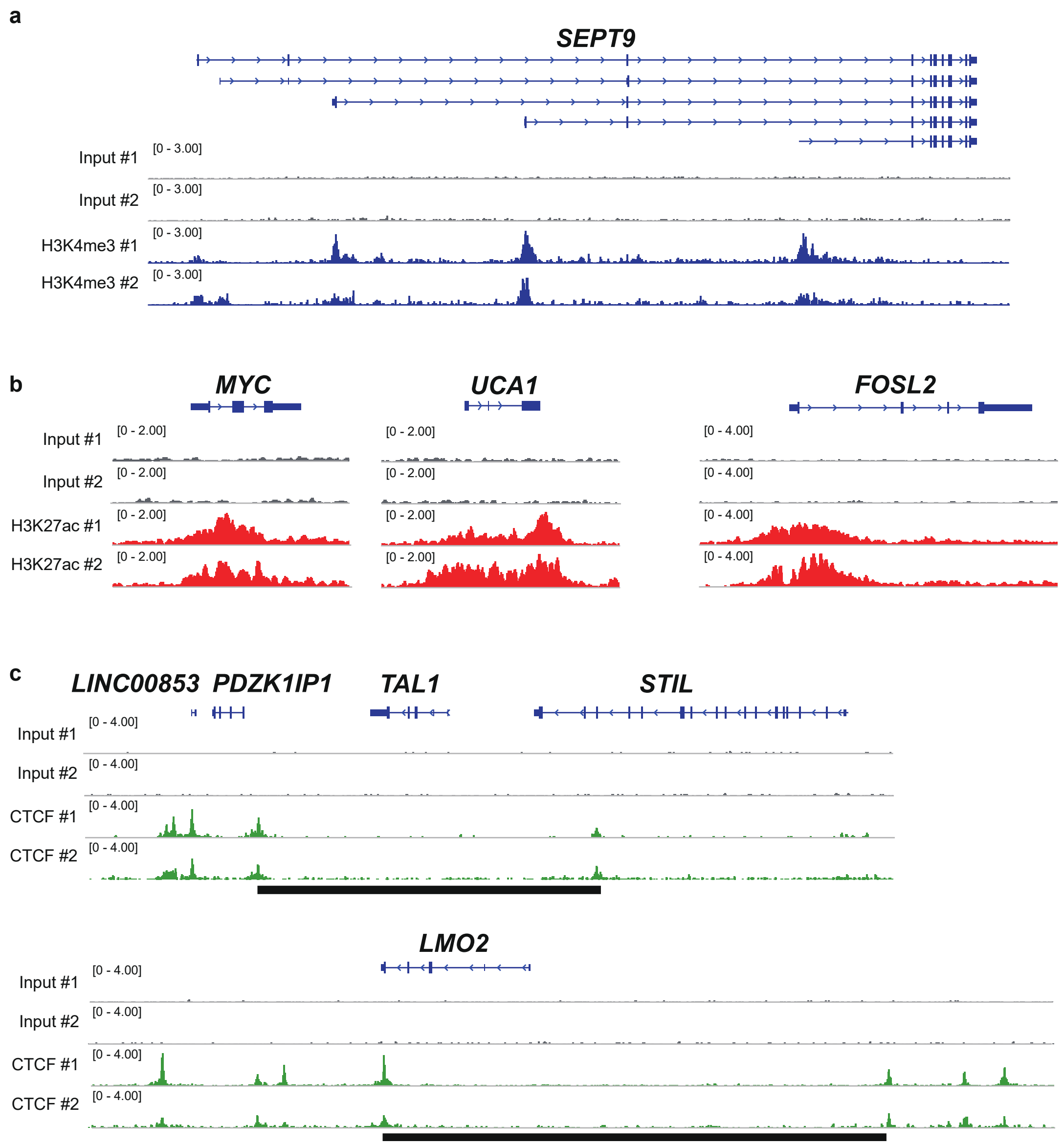

Figure S3. Tracking of epigenetic status for the individual oncogenes. (**a**) IGV tracks (blue) of H3K4me3 peaks at *SEPT9* locus as with Figure 2a. (**b**) IGV tracks (red) of H3K27ac peaks at *MYC, UCA1* and *FOSL2* loci as with Figure 2b. (**c**) IGV tracks (green) of CTCF peaks at *TAL1* and *LMO2* loci as with Figure 2c.

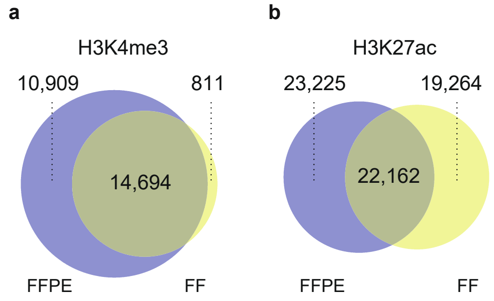

Figure S4. Comparison of ChIP-seq using FFPE and FF samples. Venn diagrams showing the overlap of H3K4me3 peaks (**a**), H3K27ac peaks (**b**) in OCCC. The total number of called peaks are shown.

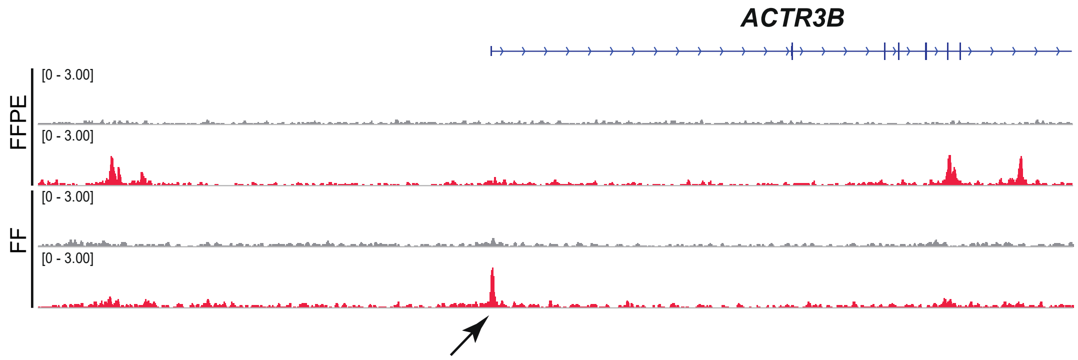

Figure S5. Examples of non-concordant peaks. Same as Figure 3b but showing the *ACTR3B* locus. Input controls are shown as grey. Non-concordant peaks are shown by the arrow.

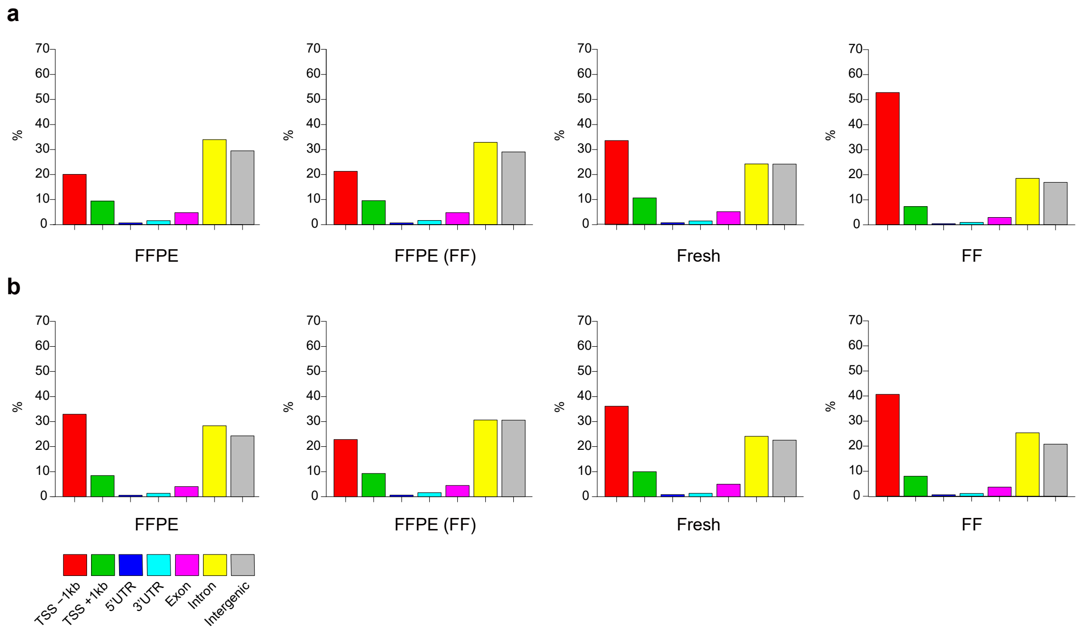

Figure S6. Bar graphs display the genomic distribution of the CTCF binding sites in FPPE, pre-frozen FFPE; FFPE (FF), Fresh, and FF tissues of endometrial endometrioid adenocarcinoma **(a)** and cervical carcinosarcoma **(b)**.

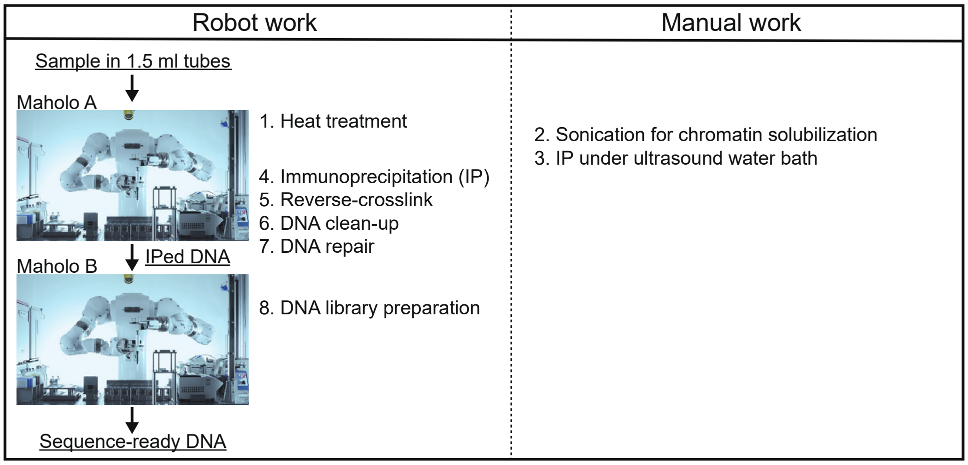

Figure S7. Procedure for the Maholo ChIP experiment. Schematic diagram of the FFPE ChIP-seq procedure implemented in Maholo. The contents of the robot's works and manual works were described. Two robots, Maholo A and B, independently performed FFPE ChIP and DNA library preparation, respectively.

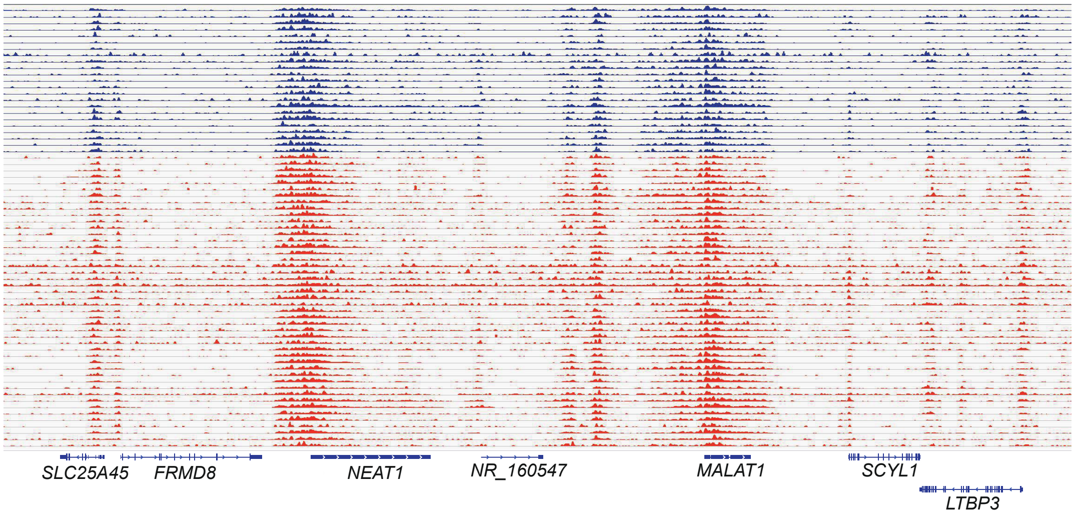

Figure S8. IGV tracks for H3K27ac ChIP-seq at NEAT1 and MALAT1 loci. EGFR-mutant positive and pan-negative cases are shown in blue and red, respectively. The data range: autoscale.

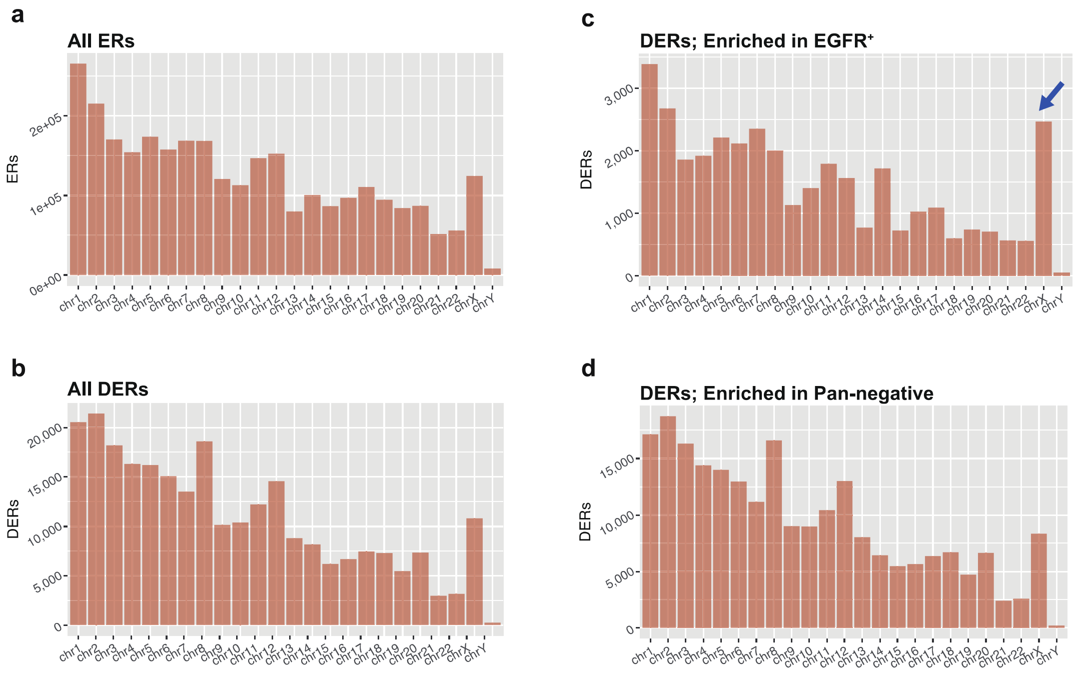

Figure S9. Chromosome distribution of H3K27ac-enriched regions. **(a)** Bar plots showing the number of enriched regions (ERs) on individual chromosomes. **(b)** Same as **a** but for differentially ERs (DERs; $|\log_2 FC| > 1$, FDR < 0.05). **(c)** Same as **b** but for DERs enriched in EGFR mutation-positive LUADs. **(d)** Same as **b** but for DERs enriched in pan-negative LUADs. The bar of X chromosome enriched in EGFR mutation-positive cases is indicated by the blue arrow.

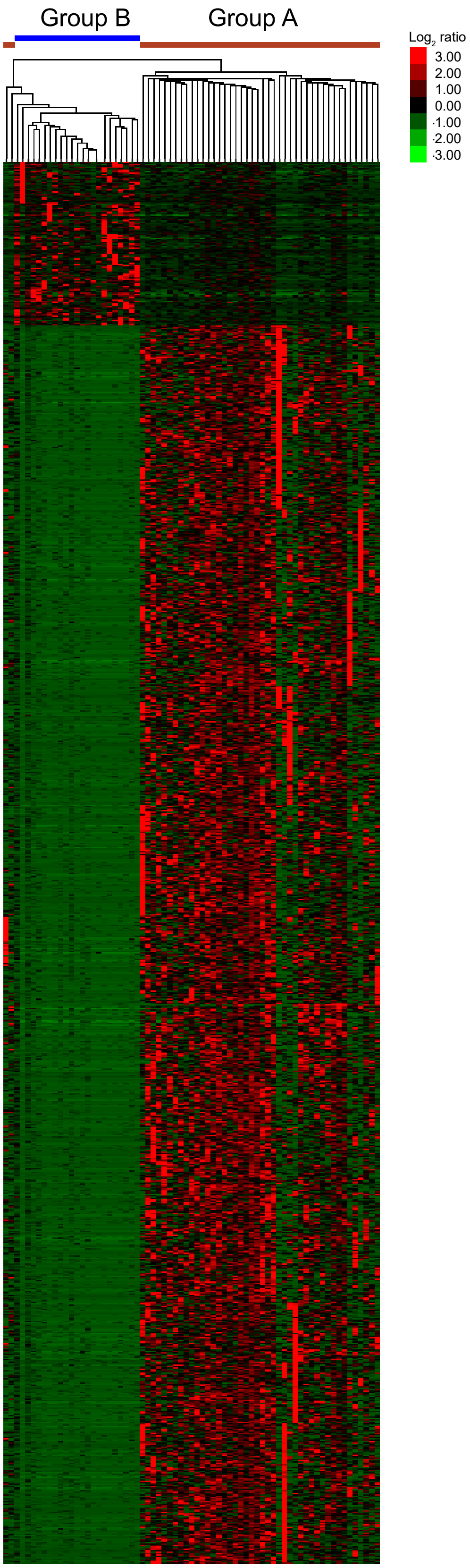

Figure S10. Majority of DERs are properly grouped. Hierarchical clustering analysis of DERs in LUADs. Clustering was performed on the 10,289 regions that were differentially enriched (|log2 FC| > 2, FDR < 5e-4) in pan-negative vs.

EGFR-mutation cases. Read counts were log-transformed to approximate a normal distribution, and each ERs was normalized by using edgeR implemented in DiffBind (see Methods). The inset legend shows the log 2-fold changes with colours (red: ERs in pan-negative, green: ERs in EGFR-mutation cases) in the heatmap. The brown (Group A) and blue (Group B) lines on the top indicate pan-negative and EGFR-mutation cases, respectively.

Table S1–3. please view at excel file.

Video S1: please view at MP4 file.
